# Effectiveness of reverse total shoulder arthroplasty for primary and secondary fracture care: mid-term outcomes in a single-centre experience

**DOI:** 10.1186/s12891-020-03903-0

**Published:** 2021-01-08

**Authors:** AM Schwarz, GM Hohenberger, M Sauerschnig, M Niks, G Lipnik, G Mattiassich, M Zacherl, FJ Seibert, M Plecko

**Affiliations:** 1 AUVA - Trauma Hospital (UKH) Styria | Graz, Teaching Hospital of the Medical University of Graz, Göstinger Straße 24, 8020 Graz, Austria; 2 Department of Orthopaedics and Trauma, Medical University of Graz, Graz, Austria

**Keywords:** Reverse total shoulder arthroplasty, Complex shoulder fracture care, Primary fracture treatment, Primary fracture reverse total shoulder arthroplasty, Secondary fracture treatment, Secondary fracture reverse total shoulder arthroplasty, Salvage procedure, Tubercle bone stock healing

## Abstract

**Background:**

The introduction of reverse total shoulder arthroplasty (RSA) as a treatment option in complex proximal humeral fractures, has significantly extended the surgical armamentarium.

The aim of this study was to investigate the mid-term outcome following fracture RSA in acute or sequelae, as well as salvage procedures. It was hypothesized that revision RSA (SRSA) leads to similar mid-term results as primary fracture treatment by RSA (PRSA).

**Methods:**

This retrospective study describes the radiological and clinical mid-term outcomes in a standardized single-centre and Inlay design. Patients who underwent RSA in fracture care between 2008 and 2017 were included (minimum follow-up: 2 years, minimum age: 60 years).

The assessment tools used for functional findings were range of motion (ROM), Visual Analogue Scale, absolute (CS) plus normative Constant Score, QuickDASH, and Subjective Shoulder Value. All adverse events as well as the radiological results and their clinical correlations were statistically analysed (using *p < .05and *95% confidence intervals).

**Results:**

Following fracture RSA, 68 patients were included (mean age: 72.5 years, mean follow-up: 46 months). Forty-two underwent primary RSA (PRSA), and 26 underwent revision RSA (SRSA). Adverse advents were observed in 13% (*n* = 9/68).

No statistically significant results were found for the scores of the PRSA and SRSA groups, while the failed osteosynthesis SRSA subgroup obtained statistically significantly negative values for ROM subzones (flexion: *p* = .020, abduction: *p* = .020). Decreased instances of tubercle healing were observed for the in PRSA group relative to the SRSA group (*p* = .006). The absence of bony healing of the tubercles was related to significant negative clinical and subjective outcomes (all scores: *p* < .05, external rotation: *p*= .019). Significant postoperative improvements were evaluated in the SRSA group (CS: 23 to 56 at mean, *p* = .001), the time from index surgery to operative revision revealed no associations in functional findings.

**Conclusions:**

RSA is an effective option in severe shoulder fracture management with predictable results for salvage as well as first-line treatment. Promising mid-term functional results, reasonable implant survival rates, and high patient satisfaction can be achieved.

**Level of evidence:**

Level III.

## Background

Proximal humeral fractures represent the third most common fracture type in the elderly population [1, 2]. The management of these injuries remains challenging due to demographic changes, as well as the simultaneous incidence of osteoporosis, and various other comorbidities [1]. The spectrum of surgical treatment modalities include closed reduction and percutaneous pin fixation [1], open reduction and internal fixation (ORIF) by locking or non-locking plates [3–7] and intramedullary nails [8, 9]. Further primary non-joint-preserving treatment options for proximal humeral fractures include anatomical total shoulder arthroplasty [10–13], shoulder hemiarthroplasty [14], and reverse shoulder arthroplasty (RSA) [13, 15–18]. However, the functional results tend to be inferior in anatomical total shoulder arthroplasty and shoulder hemiarthroplasty due to rotator cuff deficits or unsuccessful tubercle refixation in cases of fracture [10, 19, 20].

Meanwhile, recent trends show that RSA has become the treatment of choice for complex proximal humeral fractures, especially in patients with poor bone quality [21]. The RSA was primarily designed to treat patients with massive rotator cuff defects [22–25]. However, indications have been extended to further pathologies, such as cuff tear arthropathy [26, 27], and proximal humeral fractures [17, 28] or revision arthroplasty [29]. Moreover, RSA is used as a salvage procedure in cases of symptomatic mal- or nonunions following (failed) primary osteosynthesis or shoulder hemiarthroplasty of proximal humeral fractures [1, 13, 29].

The goal in clinical practice is to perform a stable osteosynthesis after meticulous, gentle reduction without denudation of fracture fragments in complex fracture situations. Various treatment options and expectations for acute proximal humeral fractures have led to vivid discussions between surgeons. To address this focus, we hypothesized that a failed joint preserving strategy treated by revision RSA might achieve similar clinical results as an approach utilizing primary joint replacement. Therefore, the aim of this study was to evaluate the functional outcome following primary RSA (PRSA) for proximal humeral fractures compared to secondary RSA (SRSA) performed as a salvage procedure.

## Methods

### Study design and patient recruitment

This retrospective case-series study characterized a single-centre evaluation in a standardized setting following reverse total shoulder arthroplasty in primary (PRSA) or secondary / sequelae (SRSA) fracture care.

**Fig. 1:**
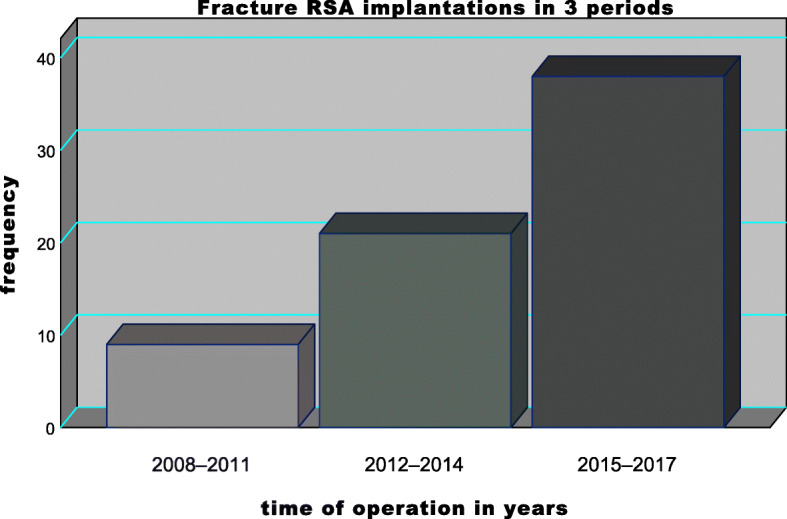
Fracture RSA implantations by years: The bar chart displays the operation time of each year in 3 time intervals. A constant increase in fracture RSA rates of implantation is shown. (RSA – reverse total shoulder arthroplasty)

All consecutive patients older than 60 years at the time of surgery were included after the treatment by either PRSA or SRSA between January 2008 and December 2017 at a level-III trauma centre (AUVA – Trauma Hospital (UKH) Styria | Graz). Figure 1 shows the years of RSA implantation (in three periods) of all included patients. The time-line was chosen based on data accessibility. Only patients with the Inlay (Grammont) design (155° humeral neck-shaft angle) were included; two different implants were involved {Delta Xtend, DePuy Synthes; Warsaw, USA and Anatomical Reverse, Zimmer; Warsaw, USA}.

**Fig. 2:**
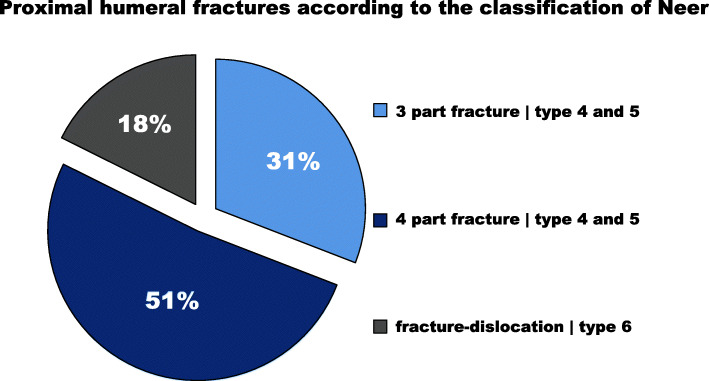
Fracture classification: The circle chart reports the distribution in percentages of all included patients based on the classification proposed by Neer. [30, 31]

**Fig. 3:**
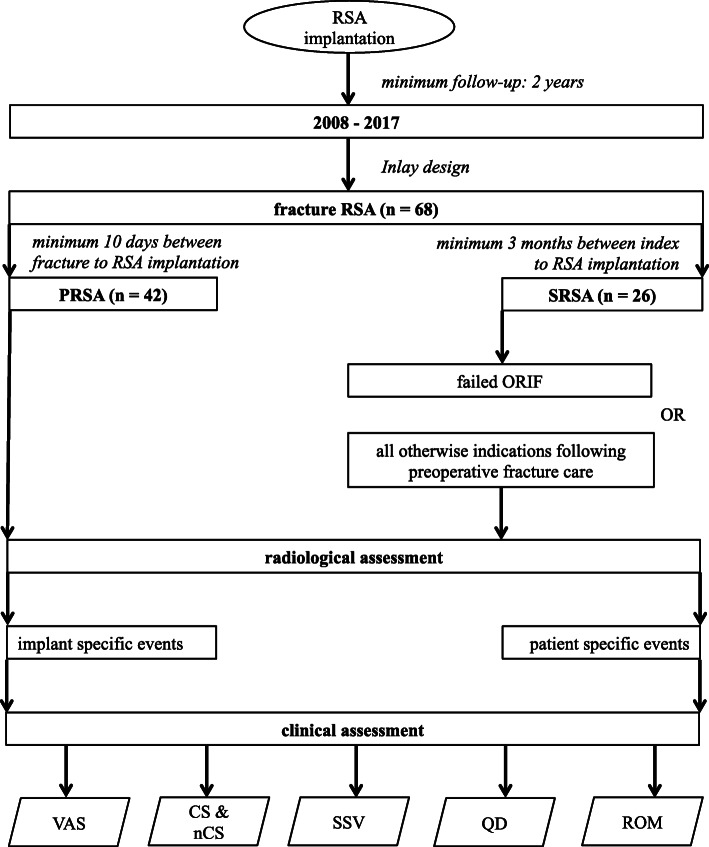
Study design: The flowchart illustrates the study focuses regarding specific target sizes and subgroup assessments. (CS – Constant score, nCS – normative Constant score, QD – QuickDash, PRSA – primary reverse total shoulder arthroplasty, ROM – Range of motion, RSA – reverse total shoulder arthroplasty, SRSA – secondary reverse total shoulder arthroplasty, SSV – Subjective shoulder value, VAS – visual analog scale)

All included patients had received RSA implantation as a primary or secondary treatment following three- or four-part fractures (types 4 and 5) and fracture-dislocations (type 6) according to the classification by Neer [30, 31]; see Fig. 2 for detailed distributions. In the PRSA group, all cases were treated with a reverse shoulder arthroplasty within ten days of trauma. For the SRSA group, a minimum time interval of three months from index to revision surgery was defined. The SRSA group was comprised of two subgroups of patients that had undergone RSA implantation in cases of failed ORIF or following all otherwise non-implant related fracture treatment adverse events. See Fig. 3 for the detailed study design.

### Patient characteristics

Data were collected prospectively in the respective hospital’s database. Patient characteristics and pathological as well as course of treatment data were collected and analysed retrospectively. All postoperative adverse events were evaluated via the hospital’s database and the patients’ medical histories were evaluated in the final patient examination. Major complications were specified as events requiring an unplanned revision, all others were classified as minor complications. The follow-up time was defined as the interval between surgery and the last assessment. The minimum follow-up was fixed at two years.

### Specific details of the operative and postoperative procedures

The operative procedure and rehab protocol were the same for the entire sample population and were in accordance with the standardized work-up at our centre. A deltopectoral approach was performed in the beach-chair position in every case. Further, all humeral monobloc components were cemented. A full 360° release was accomplished under axillary nerve visualization for glenoid preparation If necessary, an additive arthrolysis and scar release were performed.

All tubercles were fixed via non-resorbable transosseous and cerclage sutures and circular sutures around the prosthesis neck. If a detachment of the subscapularis tendon was necessary, a double-row transosseous refixation was implemented. Passive physiotherapy with a free range of motion (ROM) in a pain-free interval was started 2 days post-operation. The active-assistive motion was initiated five weeks post-operation, and deltoid muscle mass improvement was fostered at the beginning of the seventh week.

### Clinical outcome assessment

Patients were assessed to determine their current clinical level via the following scores: Visual Analog Scale (VAS), absolute Constant Score (CS) [32], normative Constant Score (nCS) [33], Subjective Shoulder Value (SSV) [34], and QuickDASH (QD) [35]. ROM was evaluated in degrees of flexion, abduction, and external rotation (ER). The internal rotation (IR) was characterized in points based on the functional shoulder-specific CS [32]. Patient-specific assessment was carried out via the modified valuation of the CS, which is based on age- and gender-related characteristics (nCS) [33].

The SSV is a Single Assessment Numeric Evaluation (SANE) of the shoulder and represents a shoulder self-assessment by the patient. The score is expressed as a percentage of an entirely healthy shoulder, which would score 100% [34]. The QD is a self-assessment instrument and includes eleven questions concerning complaints regarding the upper extremity and activities of daily living [35]. The preoperative CS, which was available in their prospective documentation in the hospital’s database, was compared to their respective postoperative values.

#### Specific targets of clinical outcomes

Further analysis of the data from the SRSA group involved considering the mid-term CS outcomes for early (< 12 months) and late (> 12 months) time intervals from index surgery to revision RSA implantation.

Additionial analysis of the entire collective regarding their CS outcomes was carried out by consideration of mid-term periods (two – five years) and longer-term periods (more than five years). See Fig. 1 for detailed RSA implantation data (displayed in years).

### Radiological outcome assessment

During follow-up, X-rays were performed in three planes (anterior-posterior, axial, and supraspinatus outlet view). The final X-rays were analysed and compared to the index and interim radiological data. These data were evaluated by three trauma and orthopaedic surgeons (AS, GH, and MN) for implant dislocation, grade of notching according to Sirveaux [36], healing of the major and/or minor tubercles, and radiological signs of loosening of the prosthesis.

**Fig. 4:**
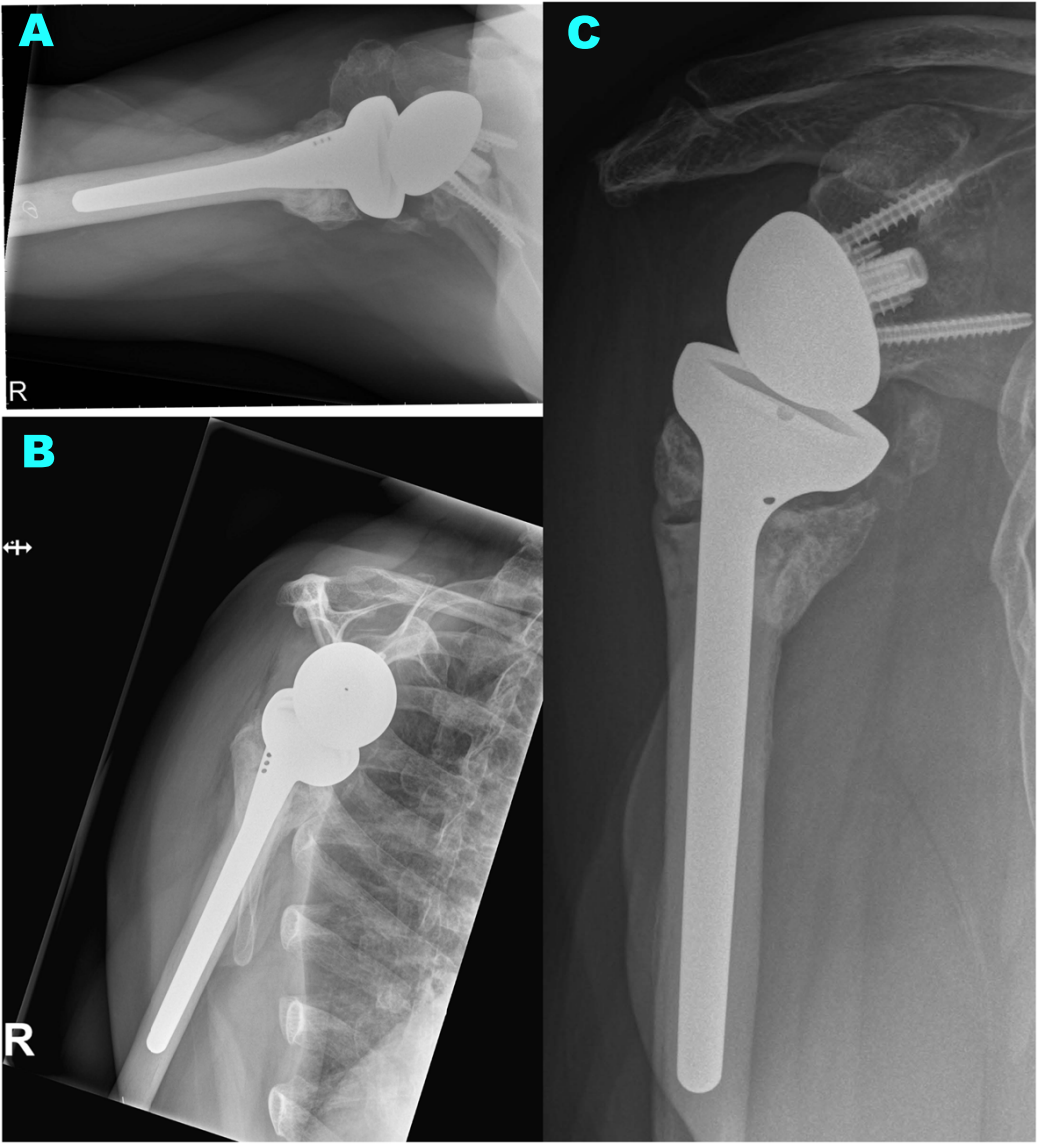
Anatomic healing of the tuberosities: A case of a 73 years old woman, an X-ray is pictured in three planes 60 months post operation. (**a**: axial view, **b**: supraspinatus outlet view, **c**: anterior-posterior view)

**Fig. 5:**
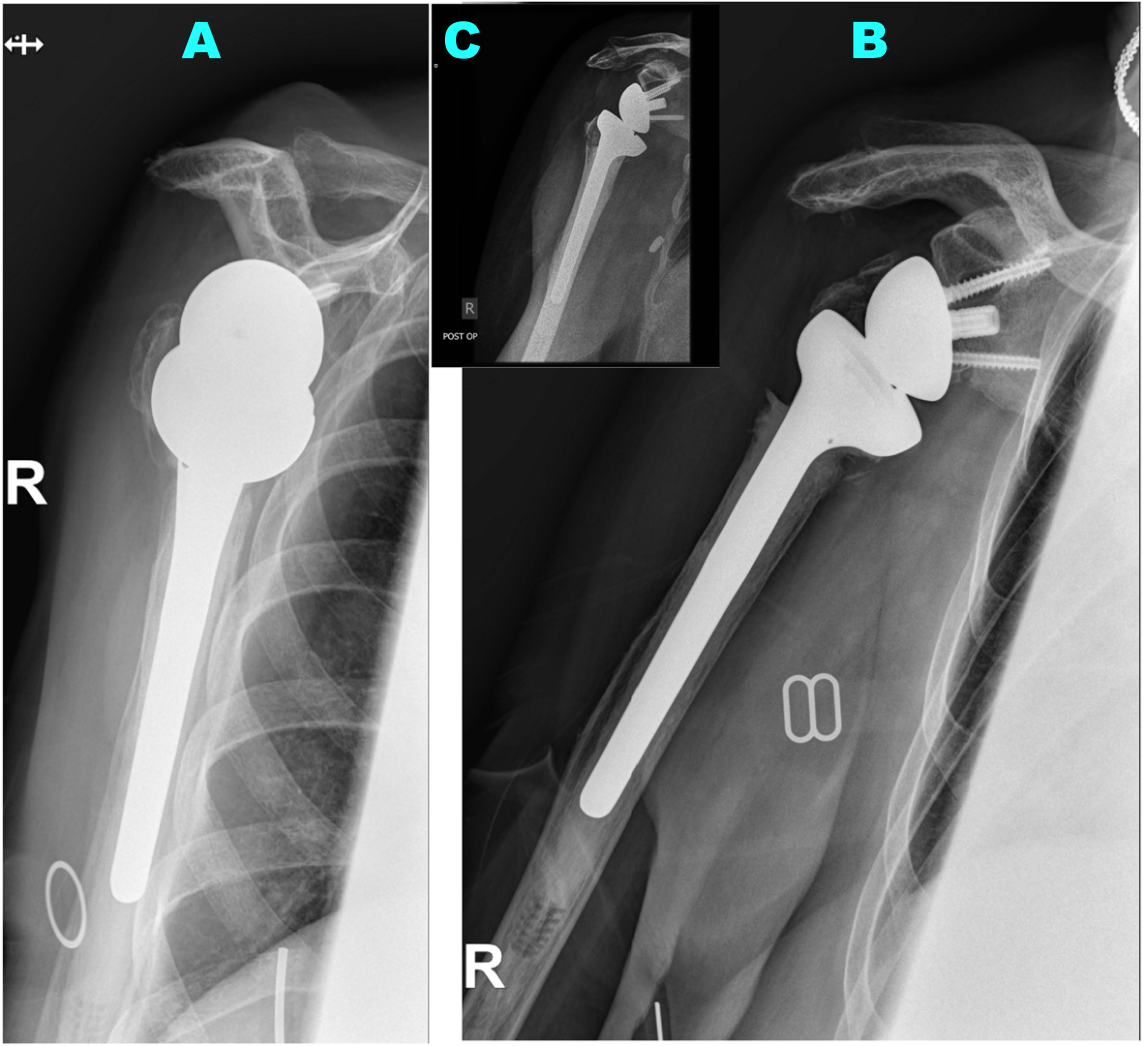
Malunion of the greater tuberosity: A case of a 78 years old woman, a X-ray is pictured in three planes 35 months post operation. A post operation index X-ray (**c**) is added for comparision of the greater tubercle to final radiological outcome. (**a**: supraspinatus outlet view, **b**: anterior-posterior view, **c**: anterior-posterior view in X-ray two days post operation)

Anatomic tubercle healing was defined by visible tubercles on the lateral and anterior part of the stem, in continuity with the diaphysis and at the level below or the same level as the top of the humeral implant (see Fig. 4). Non-anatomic healing was specified as malunion, nonunion, or resorption (see Fig. 5).

All radiological analyses were defined via raters’ consensus, which means a full agreement of all three observers in all target sizes. To assess the clinical correlations with the radiological outcomes, the CS and nCS were chosen for evaluation in the following focuses: tubercle bone stock healing and scapular notching.

### Statistical analysis

Statistical analysis was performed using the software SPSS {IBM SPSS Statistics version 26, Armonk, USA}. Continuous parameters are presented as means, standard deviations (SD), and categorical or quantitative data.

In order to compare findings between the PRSA and SRSA groups, a t-test was utilized. The Kruskal-Wallis and post-hoc Dunn-Bonferroni methods were used to interpret the data between the PRSA and both SRSA subgroups – see Fig. 3 for an illustration of the study design). The Spearman’s Rho correlation coefficient (ρ*)* was additionally used for the final relationship analyses. A Fisher’s exact test was utilized to compare the rates of adverse events. P-values (*p*) below 0.05 were considered as statistically significant, and confidence intervals of 95% were computed.

## Results

### Demographics

In total, 68 patients met the inclusion criteria. In 58 cases a Delta Xtend {DePuy Synthes; Warsaw, USA} system was utilized, and in ten cases an Anatomical Reverse {Zimmer; Warsaw, USA} system was utilized. The PRSA group consisted of 42 patients, and the SRSA group consisted of 26 patients. The SRSA collective was comprised of ten patients (39%, *n* = 10/26) who were treated following failed ORIF in the first subgroup. The second subgroup (61%, *n* = 16/26) consisted of twelve cases (46%, *n* = 12/26) who were treated after humeral head necrosis and four patients (15.5%, *n* = 4/26) who were undergoing primary trauma hemiarthroplasty.

The mean age of the entire collective was 72.5 years (SD: 6.8, range: 60–89) at the time of surgery; 73.6 years (SD: 6.8) in the PRSA group and 70.5 years (SD: 6.7) in the SRSA group. The average follow-up time for all patients was 46 months (SD: 25.1, range: 24–134); 41 months (SD: 21.3) in the PRSA group and 54 months (SD: 26.5) in SRSA group. The mean surgery time duration was 160 minutes (SD: 44.1) for the PRSA group, 207 minutes (SD: 38.2) in for the SRSA group, and 178 minutes (SD: 47.6, range 86–352) for all patients. Surgical revision was performed at an average of 45.5 months (SD: 54.1, range: 3.4–180) following index surgery in the SRSA group.

### ROM

**Fig. 6:**
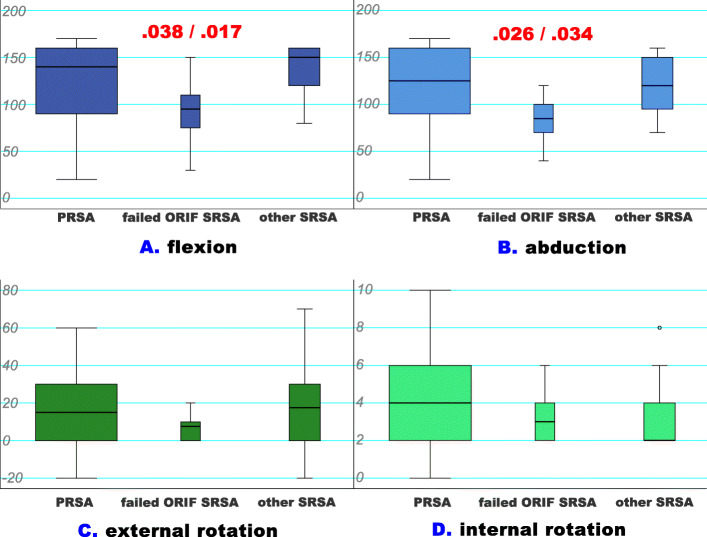
**a-d** - ROM analyses for the PRSA and SRSA subgroups: The Kruskal-Wallis test provided strong evidence of differences between the mean ranks of the three groups in (**a**) flexion (*p* = .020) and (**b**) abduction (*p* = .020). When results were adjusted using the Dunn-Bonferroni method, there was strong evidence of a difference between failed ORIF SRSA and PRSA (*p* = .038) and failed ORIF SRSA and other SRSA (*p* = .017) in terms of flexion. Similarly, there was strong evidence of a difference between failed ORIF SRSA and PRSA (*p* = .026) and failed ORIF SRSA and other SRSA (*p* = .034) in terms of abduction. The other results show that there was no statistically significant difference between external (**c**) and internal rotation (**d**). Flexion, abduction, and external rotation are displayed in degrees and internal rotation in points according to a sub-item of the Constant score [32]. (failed ORIF SRSA – reverse total shoulder arthroplasty following failed open reduction and internal fixation, PRSA – primary reverse total shoulder arthroplasty, ROM – range of motion, SRSA – secondary reverse total shoulder arthroplasty)

All average ROM values were comparable in each of the groups. No statistically significant diffference was found between the PRSA group versus the SRSA group (all P-values ≥ 0.05). When we compared the SRSA subgroups and the PRSA group, statistically significant negative average flexion (*p* = .020) and abduction (*p* = .020) values were found for patients who were previously treated following failed ORIF. The adjusted post-hoc test validated these significant results. All specific ROM values are discussed in Table 1; Fig. 6a-d. In analysing the ROM results, the strongest relationship was found for flexion and abduction with a correlation coefficient of ρ = 0.901 (*p* < .001).

**Table 1:** ROM of PRSA versus SRSA

ROM	PRSA	SRSA	*P*-value
Flexion	125° (SD: 39.6)	122° (SD: 35.3)	0.598
Abduction	118° (SD: 38.2)	107° (SD: 32.3)	0.184
ER	17° (SD: 18.7)	14° (SD: 17.8)	0.552
IR	4 (SD: 2.6)	3 (SD: 1.9)	0.169

No statistically relevant difference was observed between the PRSA and SRSA groups; good results were obtained for both groups. Flexion, abduction, and ER are reported in degrees, and IR in points according to a sub-item of the Constant Score [32]. The average values are presented

(*ER* external rotation, *IR *internal rotation, *ROM *range of motion, *PRSA *primary reverse total shoulder arthroplasty, *SRSA *secondary reverse total shoulder arthroplasty, *SD *standard deviation)

### Scores

**Fig. 7:**
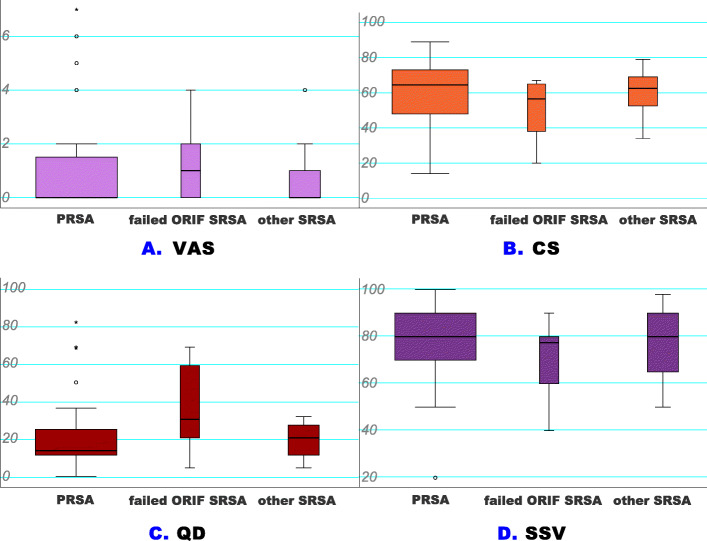
**a-d** - Score analyses for the PRSA and SRSA subgroups: The subgroup differentiation revealed no statistical significance and comparable results in all scores (Kruskal-Wallis test and Dunn-Bonferroni method: *p* ≥ .05). The SSV is displayed as percentages; all other scores are in points. (CS – Constant Score (**b**), PRSA – primary reverse total shoulder arthroplasty, QD – QuickDASH (**c**), PRSA – primary reverse total shoulder arthroplasty, SRSA – secondary reverse total shoulder arthroplasty, SSV – Subjective Shoulder Value (**d**), VAS – Visual Analog Scale(A))

The outcome values of the PRSA and SRSA groups did not reveal any statistically significant differences, as displayed in Table 2. Thus, no significant differences were observed in CS (*p* = .204) and nCS (*p* = .211). Similarly, no significant associations were found for both scores when comparing the values of the PRSA group with the values of the individual SRSA subgroups (see Fig. 7b for CS).

**Table 2:** Scores of PRSA versus SRSA

SCORES	PRSA	SRSA	*P*-value
VAS	1.1 (SD: 2.0)	0.9 (SD: 1.3)	0.573
CS	60 (SD: 16.7)	56 (SD: 15.1)	0.204
nCS	74 (SD: 19.7)	69 (SD: 18.1)	0.211
QD	23 (SD: 20.7)	25 (SD: 16.7)	0.291
SSV	76% (SD: 18.7)	75% (SD: 15.1)	0.558

Similar results without statistical significance were found for all scores. The SSV is displayed as percentages, and all other scores are shown as points. The average values are presented

(*CS* absolute Constant Score, *nCS *normative Constant Score, *PRSA* primary reverse total shoulder arthroplasty, *QD *QuickDASH, *SD *standard deviation, *SRSA * secondary reverse total shoulder arthroplasty, *SSV *Subjective Shoulder Value, *VAS* Visual Analog Scale)

When comparing the VAS and QD scores for primary and secondary care, homogeneous results were observed for the PRSA and SRSA groups (*p* = .573 and 0.291), as shown in Table 2. No statistically significant results were found regarding the VAS and QD scores for the SRSA subgroups (*p* ≥ .05, see Fig. 7a and c). Equivalent SSV results were found for the PRSA and SRSA groups (*p* = .558), see Fig. 7d. Of the score data analysed, the most substantial relationship was found for the CS and QD with a correlation coefficient of ρ = − 0.785 (*p* < .001).

**Fig. 8:**
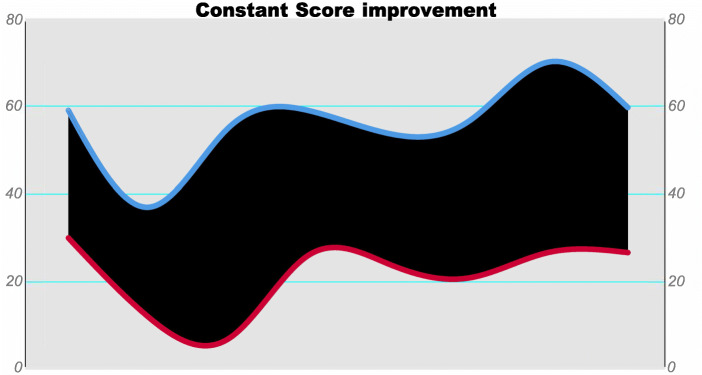
CS improvement for the SRSA group: The preoperative values to the final follow-up values are displayed in an area graph plot. The red line represents the preoperative values, and the blue line represents the postoperative values of the final control examination. A significant improvement is shown, *p* = .001. (CS – absolute Constant Score, P = P-value, SRSA – secondary reverse total shoulder arthroplasty)

A significant improvement in CS was observed in the SRSA group; the preoperative mean CS was 23 (SD: 9.7), and the postoperative average CS was 56 (SD: 13.9) in the final follow-up (*p* = .001), see Fig. 8. Additionally, SRSA differentiation in the early (< 12 months) and late (> 12 months) time intervals of operative revision were not statistically significant; all scores were comparable (*p* ≥ .05) – see Table 3. Further analysis of follow-ups in the mid-term (two - five years) and the long-term (more then five years) did not yield statistically significant results (*p* ≥ .05) – see Table 4.

**Table 3:** Clinical outcomes of early versus late times from index to SRSA surgery

SCORES	< 12 MONTHS	> 12 MONTHS	*P*-value
VAS	1.0 (SD: 1.2)	0.9 (SD: 1.4)	0.885
CS	55 (SD: 14.1)	57 (SD: 16.1)	0.831
nCS	68 (SD: 16.8)	69 (SD: 19.4)	0.883
QD	27 (SD: 21.7)	23 (SD: 13.1)	0.533
SSV	75% (SD: 16.0)	76% (SD: 14.5)	0.882

No statistical relevance was observed; good results were observed in both groups. The SSV is displayed as percentages, and all other scores are shown as points. The average values are presented

(*CS *Constant Score, *nCS *normative Constant Score, *QD *QuickDASH, *RSA* reverse total shoulder arthroplasty, *SD *standard deviation, *SRSA *secondary reverse total shoulder arthroplasty, *SSV *Subjective Shoulder Value, *VAS *Visual Analog Scale)

**Table 4:** Clinical outcomes of longer-term versus mid-term follow-ups

SCORES	2008–2013	2014–2017	*P*-value
VAS	1.3 (SD: 2.1)	0.8 (SD: 1.5)	0.319
CS	55 (SD: 18.6)	62 (SD: 14.3)	0.108
nCS	67 (SD: 22.4)	75 (SD: 17.2)	0.103
QD	27 (SD: 22.7)	21 (SD: 16.3)	0.296
SSV	75% (SD: 19.2)	77% (SD: 16.0)	0.612

No statistical relevance was observed; good functional results were found in both groups. The SSV is displayed as percentages; all other scores are in points. The average values are presented

(*CS *Constant Score, *nCS *normative Constant Score, *QD *QuickDASH, *RSA *reverse total shoulder arthroplasty, *SD* standard deviation, *SRSA *secondary reverse total shoulder arthroplasty, *SSV *Subjective Shoulder Value, *VAS *Visual Analog Scale)

### Radiological findings and their functional correlations

No implant dislocations and no glenoid loosenings were observed. In one patient, who suffered from rheumatoid arthritis, a loosening of the humeral component had to be evaluated.

Radiographic changes of the tubercles were observed in total in 28% (*n* = 19/68) of patients. A significant increase was observed in the PRSA group in comparison with the SRSA group (40% {*n* = 17/42} vs. 8% {*n* = 2/26}; *p* = .006, *ρ* = − 0.329). See Table 5 for full details. When comparing the healed with unhealed tubercles, statistically significant superiorities were documented in anatomically healed cases in all scores. (all: *p* = < 0.05). The ROM analysis verified a significant negative ER in unhealed cases (*p* = .019); see Table 6 for details.

**Table 5:** Radiographic tubercle changes in the PRSA and SRSA groups

LOCALISATION	PRSA	SRSA	*P*-value
Major tubercle	31% (*n* = 13/42)	11% (*n* = 2/26)	
Minor tubercle			
Both tubercles	9% (*n* = 4/42)		
TOTAL	*40% (n = 17/42)*	*8% (n = 2/26)*	*0.006*

Statistically significant positive tubercle changes were observed in the PRSA group; radiological changes included dislocations and (partial) resorptions in this group. In the SRSA group, only postoperative partial resorptions were detected. Significant values are shown in bold

(*PRSA* primary reverse total shoulder arthroplasty, *SD* standard deviation, *SRSA* secondary reverse total shoulder arthroplasty)

**Table 6:** – Clinical outcomes of healed versus unhealed tubercles

SCORES and ROM	HEALED	UNHEALED	*P*-value	
VAS	0.7 (SD: 1.3)	1.6 (SD: 2.5)		0.162
CS	**62 (SD: 14.1)**	**52 (SD: 19.5)**		0.029
nCS	**75 (SD: 17.3)**	**64 (SD: 22.7)**		0.021
QD	**19 (SD: 13.4)**	**34 (SD: 25.4)**		0.002
SSV	**79% (SD: 14.4)**	**68% (SD: 21.0)**		0.017
Flexion	129° (SD: 36.4)	114° (SD: 39.6)		0.125
Abduction	118° (SD: 37.7)	107° (SD: 35.3)		0.245
ER	**20° (SD:19.1)**	**8° (SD: 15.8)**		0.019
IR	4 (SD: 2.2)	3 (SD: 2.6)		0.355

Significant positive values were observed for healed tubercles. Flexion, abduction, and ER are displayed in degrees, and IR is reported in points according to a sub-item of the Constant Score [32]. The SSV is displayed in percentages, all other scores are reported in points. Significant results are marked in bold. The average values are presented

(*CS *Constant Score, *ER *external rotation, *IR *internal rotation, *nCS *normative Constant Score, *QD *QuickDASH, *ROM *range of motion, *SD *standard deviation, *SSV * Subjective Shoulder Value, *VAS *Visual Analog Scale)

A total rate of scapular notching of 23% (*n* = 16/68) was found without any correlation in the PRSA or SRSA groups (*p* = .687, ρ = 0.70; see Table 7). Only one case was classified with a higher grade of notching (grade 3 / PRSA group). No influence of postoperative CS was observed in correlation to notching cases. A mean CS of 55 was evaluated in the notching group. Patients without notching had a CS of 60 on average (*p* = .352). Similar respective values were shown for in mean nCS and notching versus non-notching: 69 versus 73 (*p* = .268).

**Table 7:** Grade of scapular notching in the PRSA and SRSA groups

NOTCHING	PRSA	SRSA	*P*-value
Grade 1	14% (*n* = 6/42)	12% (*n* = 3/26)	0.598
Grade 2	5% (*n* = 2/42)	15% (*n* = 4/26)	0.684
Grade 3	2% (*n* = 1/42)		
Grade 4			
In total	21% (*n* = 9/42)	27% (*n* = 7/26)	0.687

Similar notching rates were observed in both groups; this finding was not statistically significant

(*PRSA* primary reverse total shoulder arthroplasty, *SD* standard deviation, *SRSA* secondary reverse total shoulder arthroplasty)

### Complications

The overall rate of adverse events was 13% (*n* = 9/68). Six percent (*n* = 4/68) were classified as major, and seven percent (*n* = 5/68) as minor complications. There were fewer complications in the SRSA group when compared with the PRSA group, but this result was not statistically significant (*p* = .196). The seven complications (in six patients) in the PRSA group included two patients requiring revision surgery. This represents a rate of adverse events of 17% in the PRSA group (*n* = 7/42). The two complications in the SRSA group were a single major and minor complication, which led into one revision surgery. Hence, a rate of 8% (*n* = 2/26) was observed in the SRSA group. The details of all adverse events are listed in Table 8.

**Table 8:** Adverse events

	EVENT	MONTHS	INTERVENTION	CLASSIFICATION
1	spontaneous muscle hematoma (under direct *oral anticoagulant*)	60	conservative	**minor**
2	spontaneous muscle hematoma (under direct *oral anticoagulant*)	35	conservative	**minor**
3	instability with dislocation	10	inlay change	**major**
4	major tubercle impingement	7	conservative	**minor**
5	traumatic periprosthetic fracture (accident)	131	ORIF	**major**
6	scapular spine fracture	116	conservative	**minor**
7	shaft loosening	70	conservative (non vult)	**major**
8	traumatic periprosthetic fracture (fall)	25	ORIF	**major**
9	plexus neuropraxia	perioperative	conservative	**minor**

Major and minor complications of total follow-ups in all patients are displayed. Their time- and event-related specifications are shown. The time from RSA implantation to each adverse event is presented in months. The events numbered from 1 to 7 involved PRSA patients, and events numbered from 8 to 9 involved SRSA patients. Multiple events are possible in one patient

(*ORIF *open reduction and internal fixation, *PRSA *primary reverse total shoulder arthroplasty, *RSA *reverse total shoulder arthroplasty, *SRSA *secondary reverse total shoulder arthroplasty)

## Discussion

The aim of the study was to analyse the effectiveness of the RSA for fracture care in mid-term outcomes via a standardized setting. While RSA significantly improved the treatment of patients with rotator cuff disorders, we focused on the value and better understanding of RSA in fracture management.

The PRSA and SRSA groups did not show statistically significant differences in the range of motion and functional scores, which confirms our hypothesis. Only the subgroup following failed ORIF showed significantly lower values for flexion and abduction when compared with the PRSA group.

Significant improvements between pre- and postoperative CS could be observed in the SRSA group. Furthermore, the time from index to revision surgery had no impact on the functional outcome. Similary, in assessing the longer versus shorter follow-up of the entire collective, no statistically significant decrease in functional findings was observed. These facts indicate that RSA implantation represents a successful treatment strategy in fracture (salvage) care following our standardized operative and postoperative protocol. However, radiographic changes in the tubercles and the overall complication rate were higher in the PRSA group when compared with the SRSA group.

Grubhofer et al. [37] evaluated 51 patients who had undergone RSA for complex proximal humeral fractures with an average follow-up of 35 months. At the final follow-up check, the absolute CS was at a mean of 62 points, which is well comparable to the PRSA group of our sample (at mean: 60 points).

The French Society of Orthopaedic and Traumatology Surgery [38] performed a pro- and retrospective multicentre study involving nine institutions to investigate RSA outcomes in patients with four-part proximal humeral fractures. The retrospective part of the study included 41 patients with a mean follow-up of 39 months, and the prospective part involved 32 patients with an average follow-up of eleven months. The mean absolute CS was 57 (retrospective) and 50 (prospective) points, respectively. Both values are comparable to our PRSA group (average CS: 60).

Grubhofer and colleagues [39] evaluated 44 shoulders that had undergone revision RSA following unsatisfactory outcomes after proximal humeral ORIF at a mean follow-up of 46 months. The authors reported a statistically significant improvement in CS (pre-RSA: 26 [4–54] points; post-RSA: 55 [19–80] points). Their outcome is similar to our failed ORIF SRSA subgroup (mean CS: 52 points). Further, the significant increase of CS in our results, from a preoperative mean of 23 to a postoperative mean of 56, represents an equivalent effect.

Dezfuli et al. [40] evaluated a sample of 49 patients receiving RSA for either acute proximal humeral fracture, mal- or nonunion, failed ORIF, or trauma hemiarthroplasty. As in our sample, the authors found no statistically significant differences between the subgroups.

Cicak et al. [41] evaluated 37 patients treated with RSA for either acute proximal humeral fractures or sequelae of these. For 21 of these patients, RSA was the primary surgical treatment (14 of these had chronic fracture situations and seven had acute proximal humeral fractures). A further 16 patients had undergone previous surgical therapy, including ORIF or percutaneous fracture fixation. The group of patients that received RSA for acute fractures had a mean ER of 28° and an average IR up to the L4 level. In comparison, our PRSA sample had an average ER of 17° and a mean IR of four points (CS: at L5/S1 level) [32]. The authors reported a mean ER of 19° and an average IR to L4 for the subgroup that had undergone previous surgery. Our SRSA subgroup showed an ER of 14° on average and a mean IR of three points (CS: the level between the buttock and iliosacral joint) [32].

Grubhofer et al. [39] evaluated 44 shoulders that had received RSA due to unsatisfactory ORIF and reported an improvement of the preoperative SSV from 29% (range: 0–90%) to 67% (range: 5–95%) at the final control examination. We found almost equal values for our PRSA and SRSA groups (76% and 75%, respectively). The same authors [37] re-evaluated 51 patients (52 shoulders) treated with RSA for acute proximal humeral fractures. The authors reported a mean SSV of 83% (range: 0-100%). The French Society of Orthopaedic and Traumatology Surgery [38] reported a mean SSV of 75% for their retrospective study group and 69% for their prospective collective. These data are well comparable to our PRSA group, which showed an average SSV of 76%.

Complication rates for RSA are reported to range from 19–68% [22, 42–44], and include a high percentage of scapular notching and impairment of external rotation as the main problems [18]. Furthermore, RSA may involve periprosthetic fractures, fractures of the glenoid, acromion or humeral shaft, neurological lesions, infection, dislocation, mechanical failures or loosening of the glenosphere [2, 22]. Lehtimäki et al. [2] identified all RSAs utilized for proximal humeral fractures from the Nordic Arthroplasty Register Association registry data for the interval between 2004 and 2016, whereby 1523 implantations were included in the study. Only 2% of these (33/1523) required revision surgery with instability reported as the most common reason (11/1523). The nine adverse effects (13%) and 6% major complication rate of the present study have to be re-evaluated. Implant-related major complications were present in only one case and were soley due to instability. A shaft loosening had to be attributed to an underlying disease. All other adverse events were not implant-related or fateful events. These facts lead to a major complication rate of 3% in our total collective, comparable to the findings of Lehtimäki et al. [2] in a sample with a high number of included patients. Moreover, the rate of adverse events was similar in primary and secondary care.

Common agreements exist regarding decreasing notching rates in modern prosthesis designs [45]. The clinical impact of scapular notching appears to be controversial in the literature. Some authors report a significant decrease in outcomes by notching; others declare no significant influence [46–48]. A recent systematic review [49], including 2,222 shoulder arthroplasties, found that 155° implants had a total notching rate of 16.8%. The authors reported that the notching rate was significantly higher in the 155° design than in the 135° design. These values are comparable to our results for a 23% notching rate.

Jain et al. [50] identified a tubercle healing rate of 70.5%, which is equivalent to our results of 72% in a meta-analysis of 382 shoulder arthroplasties and a similar follow-up period. Acceptable functional results in unhealed tubercle patients (28% of the total collective) substantiate that a fracture RSA implantation allows predictable postoperative results. Nevertheless, statistically significant superiorities for healed tubercles were shown in all functional scores and patient satisfaction characteristics, as well as in ER – an essential aspect for the daily processes. Based on these data for mid-term clinical outcomes, we recommend that surgeons focus on tubercle refixation.

### Strengths and limitations

This study investigated the combined effect of radiological events and tubercle bone stock associated with clinical outcomes in primary and secondary fracture care and allowed preoperative assessments in order to estimate postoperative processes. Due to changes in patient requirements and the increasing demand for salvage procedures, this study reinforces our understanding with mid-term results in an assimilable patient number. The clinical significance is fortified by the continuously increasing implantation rates of RSA in fracture care in modern material designs. Future research is needed to establish potential superiorities and the best surgical options for younger patients.

However, there are several limitations to this study. First, the study design was retrospective in nature, and our preoperative documentation did not collect all the tests and scores used in our follow-up study (with the exception of the SRSA group). Therefore, amelioration only applies to the CS and nCS in the SRSA group.

Second, our follow-up period was limited, and the complication rate might increase with time. Next, group sizes in number of patients, implant design and follow-up varied. This aspect is comparable to the literature data in this patient sample. Furthermore, the study was conducted using only reverse total shoulder systems with cemented humeral components, and direct comparisons to their contemporaries the cementlesses RSA systems, cannot be drawn. Lastly, multiple surgeons (four experienced trauma and orthopaedic surgeons) were involved, and no comparisons regarding their personal experiences and outcomes were performed.

## Conclusions

The similar mid-scale/-term findings of both strategies confirm the value of RSA in complex shoulder fracture management as primary or secondary / salvage care to maintain autonomy. Predictable, promising mid-term functional results, high patient satisfaction, and excellent pain relief could be demonstrated.

In SRSA, significant postoperative improvements could be observed. The time from index surgery to operative revision was not a no statistically significant factor for clinical outcomes.

A satisfactory total tubercle healing rate was proven, since a significant increase of radiographic changes was shown in the PRSA group. Statistically significant improvements in objective and subjective findings for healed tubercles were shown. Further, no statistically significant correlations were observed for notching in the PRSA or SRSA groups in the modern material design.

In our setting, RSA has become a successful and effective option for complex proximal humeral cases with inadequate bone stock. This non-joint-preserving management burdened by a low complication rate and a reasonable implant survival rate, whereby a specific patient selection should occur due to the few available options in cases of RSA failure.

## Data Availability

The datasets used and/or analysed during the current study are available from the corresponding author upon reasonable request.

## Abbreviations

AUVA: Austrian Social Insurance for Occupational Risks
CS: absolute Constant Score
ER: external rotation
IR: internal rotation
nCS: normative Constant Score
ORIF: open reduction and internal fixation
QD: QuickDASH
*p*: *p*-value
PRSA: primary reverse total shoulder arthroplasty
ρ: Spearman's Rho correlation coefficient
ROM: range of motion
RSA: reverse total shoulder arthroplasty
SANE: Single Assessment Numeric Evaluation
SD: standard deviation
SRSA: secondary reverse total shoulder arthroplasty
SSV: Subjective Shoulder Value
VAS: Visual Analog Scale
